# The analysis of immunogenic cell death induced by ablation at different temperatures in hepatocellular carcinoma cells

**DOI:** 10.3389/fcell.2023.1146195

**Published:** 2023-04-28

**Authors:** Mengdong Wang, Yaxin Duan, Mao Yang, Yongfei Guo, Fengtan Li, Junping Wang, Tongguo Si

**Affiliations:** ^1^ Department of Radiology, Tianjin Medical University General Hospital, Tianjin, China; ^2^ Key Laboratory of Cancer Prevention and Therapy, Department of Interventional Treatment, National Clinical Research Center for Cancer, Tianjin’s Clinical Research Center for Cancer, Tianjin Cancer Hospital Airport Hospital, Tianjin Medical University Cancer Institute and Hospital, Tianjin, China

**Keywords:** ablative therapy, apoptosis, calreticulin, extracellular ATP, CXCL10

## Abstract

**Introduction:** Ablation therapy is a commonly used tool in the management of hepatocellular carcinoma (HCC). After ablation, dying cancer cells release a variety of substances that trigger subsequent immune responses. Immunogenic cell death (ICD) has been a trending topic in recent years and has been discussed many times along with oncologic chemotherapy. However, the subject of ablative therapy and ICDs has been little discussed. The purpose of this study was to investigate whether ablation treatment induces ICD in HCC cells and whether different types of ICDs arise because of different ablation temperatures.

**Methods:** Four different HCC cell lines (H22, Hepa-16, HepG2 and SMMC7221) were cultured and treated under different temperatures (−80°C, −40°C, 0°C, 37°C, and 60°C). Cell Counting Kit-8 assay was performed to analyze the viability of different cell lines. Apoptosis was detected by flow cytometry assay, and a few ICD-related cytokines (calreticulin, ATP, high mobility group box 1, and CXCL10) were detected by immunofluorescence or enzyme-linked immunosorbent assay.

**Results:** The apoptosis rate of all kinds of cells increased significantly in −80°C group (*p* < 0.01) and 60°C group (*p* < 0.01). The expression levels of ICD-related cytokines were mostly significantly different between the different groups. For calreticulin, Hepa1-6 cells and SMMC7221 cells showed significantly higher protein expression levels in 60°C group (*p* < 0.01) and significantly lower protein expression levels −80°C group (*p* < 0.01). The ATP, high mobility group box 1 and CXCL10 expression levels were significantly higher in 60°C, −80°C and −40°C group of all four cell lines (*p* < 0.01).

**Conclusion:** Different ablative treatments could induce different types of ICDs in HCC cells, providing a promising track for the development of individualized cancer therapies.

## 1 Introduction

Hepatocellular carcinoma (HCC) is one of the most common and deadliest cancers and is the third key contributor to tumor-related deaths worldwide, accounting for approximately 830,000 deaths each year (Gilles et al., 2022). There are currently four dominant therapeutic options for HCC: liver resection, liver transplantation, chemotherapy, and ablation therapy (Cui et al., 2016). As a clinically well-established local treatment, ablation therapy can be performed with various techniques. Physical techniques include heating and freezing, such as radiofrequency ablation (RFA) and cryoablation (CRA) (Bai et al., 2021). Chemical method usually uses absolute ethanol. By inducing cell necrosis, ablative therapy releases abundant cell fragments *in situ*, which expose formerly sheathed tumor antigens to the immune system and provoke a pro-inflammatory response that may result in either growth inhibition (Shibata et al., 1998) or promotion (Soanes et al., 1970) of a distant tumor. Preclinical and clinical cases have witnessed both positive (remote tumor regression) and negative (remote tumor growth) consequences post-ablation.

To unravel how the immune system deciphers the complex signals sent by dying cancer cells, considerable efforts have been devoted. Notably, a precise combination of damage-associated molecular patterns (DAMPs) presented in a spatiotemporally orchestrated sequence by dying cancer cells can bolster the immune reaction that drives specific T-cell immunity, thereby implementing durable tumor regression and facilitating immunologic memory (Takaki and Cornelis, 2018; van den Bijgaart et al., 2017).

To distinguish the different forms of dying tumor cells, a novel concept of immunogenic cell death (ICD) was introduced. Through concerted expression and release of a broad array of DAMPs, this specific form of apoptosis can lead to a pro-inflammatory immune process in an immunocompetent host (Kroemer et al., 2013). Related DAMPs mainly are calreticulin (CRT) translocated to the cell membrane, extracellular ATP, type I interferon (IFN), and high mobility group box 1(HMGB1). Besides, a number of cytokines capable of inducing activation and chemotaxis of immature immune cells, such as interleukin (IL)-1, IL-17, IFN-γ and C–X–C motif ligand 10 (CXCL10), play a role in this complex immunological process (Gordon and Martinez, 2010).

In recent years, a series of articles have explored whether the commonly used oncologic chemotherapeutic agents in clinical practice carry the ability to trigger ICDs (Pol et al., 2015; Zhou et al., 2019; Fucikova et al., 2020), while the field of ablative therapy and ICDs has been little explored. This study was initiated on a hypothesis that ablation may induce ICD in cancer cells. Through experiments performed on *in vitro* cultured HCC cells, we aimed to answer these two questions: (1) whether heat ablation or cryoablation, sparks ICDs in HCC cells, (2) whether the different temperature sparks different ICDs. We cultured two murine HCC cell lines (H22 and Hepa-16), and two human HCC cell lines (HepG2 and SMMC7221). Then we subjected them to acute cryoablation or heat ablation at different temperatures (−80°C, −40°C, 0°C, 60°C), with 37°C serving as a control group. Cell apoptosis was measured by flow cytometry, and the expression levels of various ICD-related cytokines were detected by immunofluorescence or enzyme-linked immunosorbent assay. Our data suggested that in different HCC cell lines, different treatment induced different expression levels of ICD-related cytokines.

## 2 Materials and methods

### 2.1 Cell culture and cryoablation or heat ablation implementation

The human HCC cell lines (SMMC7721, HepG2), and the mouse HCC cell lines (H22 and Hepa1-6) were kindly provided by Stem Cell Bank, Chinese Academy of Sciences. The cells were grown and maintained in RPMI-1640 medium (Sigma-Aldrich, R8758) containing 10% fetal bovine serum (FCS; Merck KGaA, Darmstadt, Germany) and antibiotics (10,000 U/mL penicillin and 10 mg/mL streptomycin), and the culture was incubated at 37°C in a 5% CO_2_ atmosphere.

For each cell line, cells were divided into five groups for different thermal-treatment, −80°C, −40°C, 0°C, 37°C, 60°C. For cryoablation group, cells were placed in a refrigerator at −80°C, −40°C, or 0°C for 30 min. For heat ablation group and control group, cells were heated in a 60°C or 37°C water bath at for 30 min.

### 2.2 Cell counting Kit-8 (CCK-8) assay

Viability of different HCC lines following freezing or heating was analyzed by CCK-8 assay according to the manufacturer’s protocols. In brief, HCC cells were cultured at a concentration of 5 × 10^5^/mL per well into 96-well culture plates. After 24 h of incubation, the cells were subjected to the above-mentioned cryoablation or heat ablation. Then, the plates were rinsed, added 10 μL of enhanced CCK-8 reagent (Vazyme, Nanjing, China) per well, and then incubated for 4 h. Lastly, the 96-well plates were scanned at a wavelength of 490 nm using a microplate reader, and cell survival rate was calculated.

### 2.3 Flow cytometry

Flow cytometry was performed using a FITC-Annexin V and PI Apoptosis kit (BioScience, China) according to the instruction. In brief, cells were collected after the ablation treatment and resuspended with cold PBS buffer and counted. 1 × 10^5^ resuspended cells of each cell line were centrifugated and gently resuspended in 195 μL Annexin V-FITC binding buffer. Next, cells were stained with 5 μL Annexin V-FITC and 10 μL propidium iodide. After 20 min of incubation at 4°C in darkness, the percentage of apoptosis rate was determined using flow cytometry (BD Canto II, United States).

### 2.4 Enzyme-linked immunosorbent assay (ELISA) analysis

To evaluate whether cryoablation or heat ablation could induce the predicted emission of specific cytokines, the levels of CXCL10, HMGB-1, and ATP were quantified with commercial ELISA kits (Biomatik, Ontario, Canada) in accordance with the manufacturer’s instructions. For each cytokine, required number of microplate strips were taken out from the matching ELISA kit, and the rest were sealed and stored at 4°C. The set standard wells were spiked with different concentrations of standards, 50 μL each. In the set sample wells, 10 μL of the sample was added firstly, and then 40 μL of sample dilution. No reagents were added to the set blank wells. Next, 100 μL of horseradish peroxidase (HRP)-labeled detection antibody was added to each well, except for the blank wells. The top of the wells was covered firmly with a seal until the end of a 60-min incubation. Then the wells were washed for 5 times and treated with 50 μL of substrate. And after a 30-min incubation in the dark for color development, each well was applied with 50 μL stop solution. The optical density was read at 450 nm using a Multiskan™ GO detector system.

### 2.5 Immunofluorescence

The expression of CRT was detected by immunofluorescence assay. Different HCC cells were grown in 24-well plates and underwent cryoablation or heat ablation. After 3 washings with PBS and a 10-min fixation with 4% paraformaldehyde, each well was permeabilized by 200 μL of 0.2% Triton X-100 for 10 min. Next, 5% BSA was added to each well for 1 h to block non-specific staining. Then the primary antibody (Sigma-Aldrich) at 1:400 dilution was applied to the wells, which were stored at 4°C overnight. Followed by 4 washings, the wells were applied with Alexa Fluor 488-labeled goat anti-rabbit IgG (H + L) antibody (Invitrogen, CA, United States) at 1:400 dilution, incubated at room temperature for 1 h and washed 4 times. Finally, 150 μL of DAPI was added to each well. After a 4-min incubation at room temperature, the plates were washed with PBS for the last 4 times. The slides were examined under a fluorescence microscope, and the fluorescence intensity of each sample was recorded.

### 2.6 Statistical analysis

Dates are presented as the mean ± standard deviation (SD). The statistical analyses were performed using SPSS 25.0 software (SPSS Inc., Chicago, United States) and figures were made by GraphPad Prism version 9 (GraphPad Software, Inc., San Diego, United States). Comparison between different groups was determined by one-way ANOVA with Turkey’s multiple comparison test. *p*-value <0.05 was considered a statistically significant difference.

## 3 Results

### 3.1 Effect of thermal ablation treatment on the viabilities of HCC cells

**FIGURE 1:**
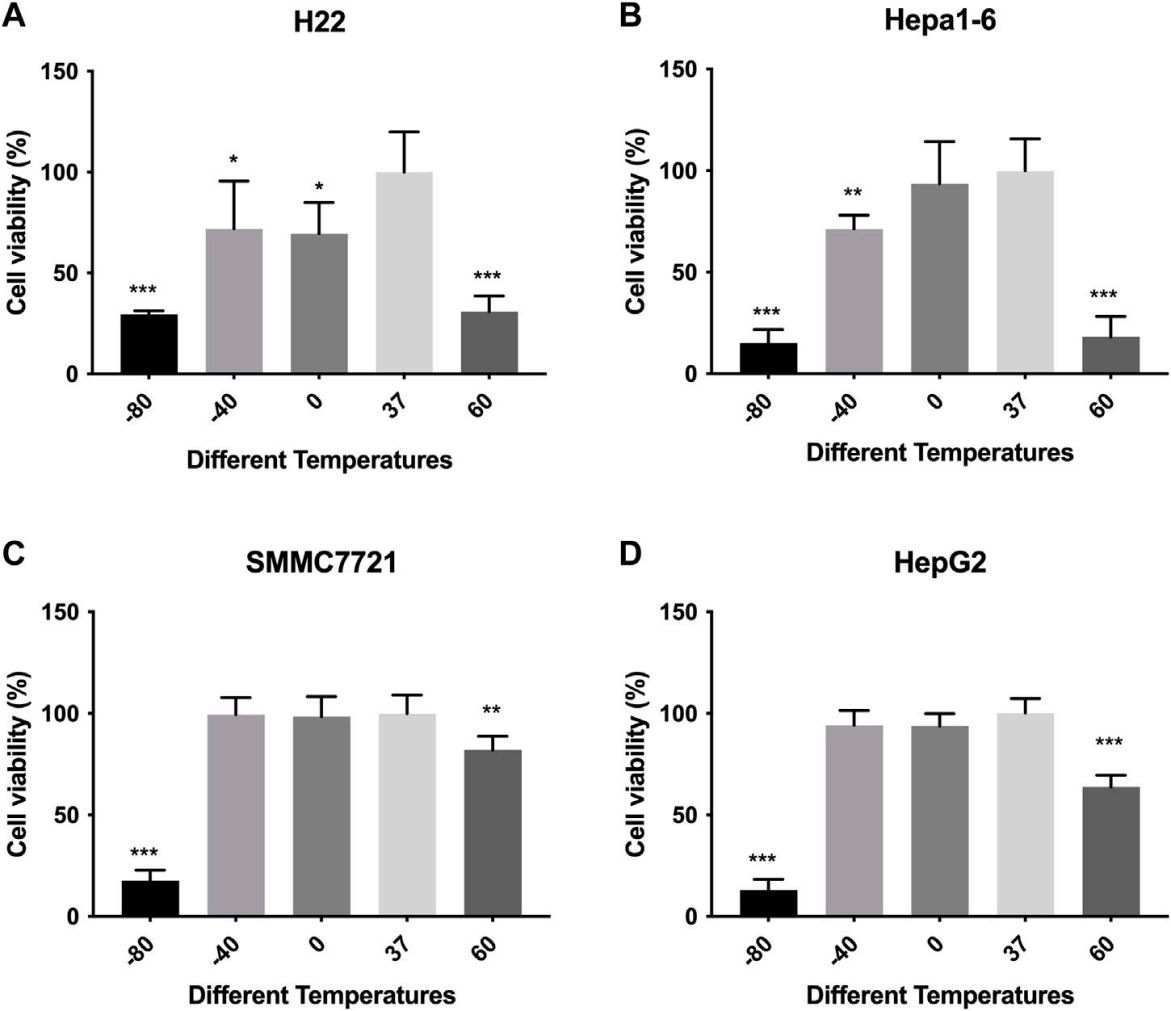
Cell viability results of **(A)** H22 cells, **(B)** Hepa1-6cells, **(C)** SMMC7721 cells, and **(D)** HepG2 cells following different thermal ablations measured by CCK8 assay (* indicates *p* < 0.05, ** indicates *p* < 0.01 and *** indicates *p* < 0.001).

Cell viabilities of different HCC cell lines were measured by CCK-8 method. The response of *in vitro* cultured HCC cells to ablation at different temperature is concluded in Figure 1. The viability of murine (H22 and Hepa-16) HCC cells decreased after treatment compared with control group, especially in −80°C (*p* < 0.01) and 60°C groups (*p* < 0.01), while no difference was observed as between −80°C and 60°C groups. The viability of human HepG2 HCC cells decreased after treatment, but the viability of SMMC7721 cells decreased obviously only in −80°C group (*p* < 0.01), not in −40°C group. And the cell viability (SMMC7721 and HepG2) in −80°C group was much lower than 60°C group (*p* < 0.01).

**FIGURE 2:**
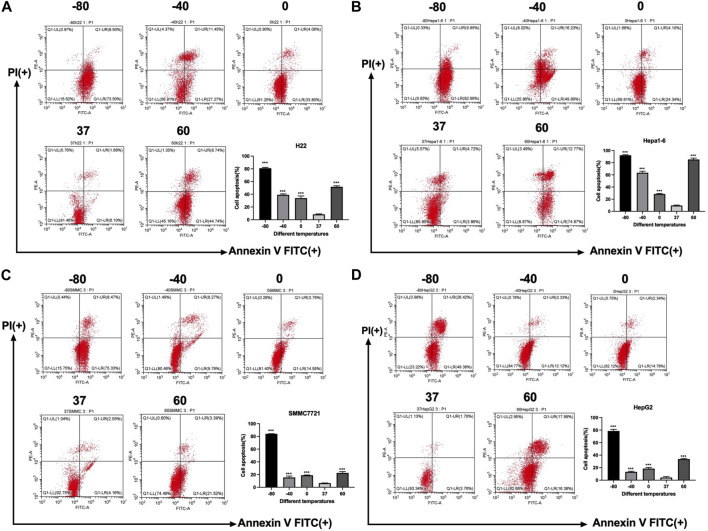
Apoptosis results of **(A)** H22 cells, **(B)** Hepa1-6cells, **(C)** SMMC7721 cells, and **(D)** HepG2 cells following different thermal ablations measured by flow cytometry (** indicates *p* < 0.01 and *** indicates *p* < 0.001.).

Furthermore, an apoptosis assay was performed by co-staining with annexin V and PI. Flow cytometry was utilized to measure the apoptosis rate of H22, Hepa1-6, SMMC7721, and HepG2 cells. As shown in Figure 2, the apoptosis rate of all kinds of cells increased significantly in −80°C group (*p* < 0.01) and 60°C group (*p* < 0.01). And for SMMC7721 and HepG2 cells, the apoptosis rate in −80°C group was much higher than 60°C group (*p* < 0.01).

### 3.2 Effect of thermal treatment on CRT levels in HCC cells

**FIGURE 3:**
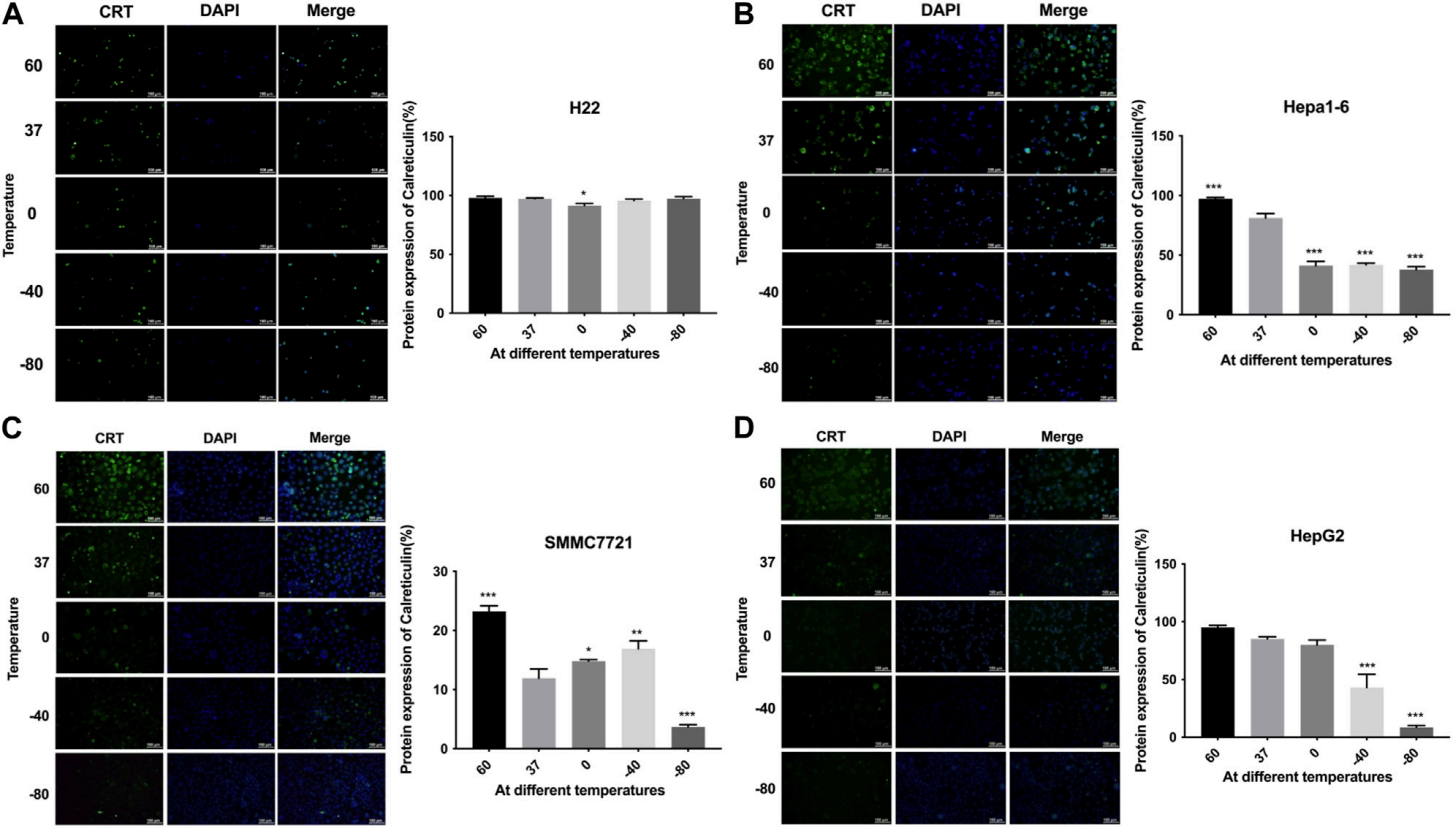
Immunofluorescence results of CRT expression of **(A)** H22 cells, **(B)** Hepa1-6cells, **(C)** SMMC7721 cells, and **(D)** HepG2 cells following different thermal ablations (* indicates *p* < 0.05, ** indicates *p* < 0.01 and *** indicates *p* < 0.001).

The expression levels of CRT were detected by cellular immunofluorescence assay. The results are shown in Figure 3. H22 cells showed no significant changes in CRT expression in −80°C group or 60°C group (*p* > 0.05). Hepa1-6 cells and SMMC7221 cells showed significantly higher protein expression levels in 60°C group (*p* < 0.01) and significantly lower protein expression levels in −80°C group (*p* < 0.01). HepG2 cells showed no significant changes in protein expression levels under 60°C conditions, but significantly lower protein expression levels in −80°C group (*p* < 0.01).

### 3.3 Effect of thermal treatment on ATP and HMGB1 levels in HCC cells

**FIGURE 4:**
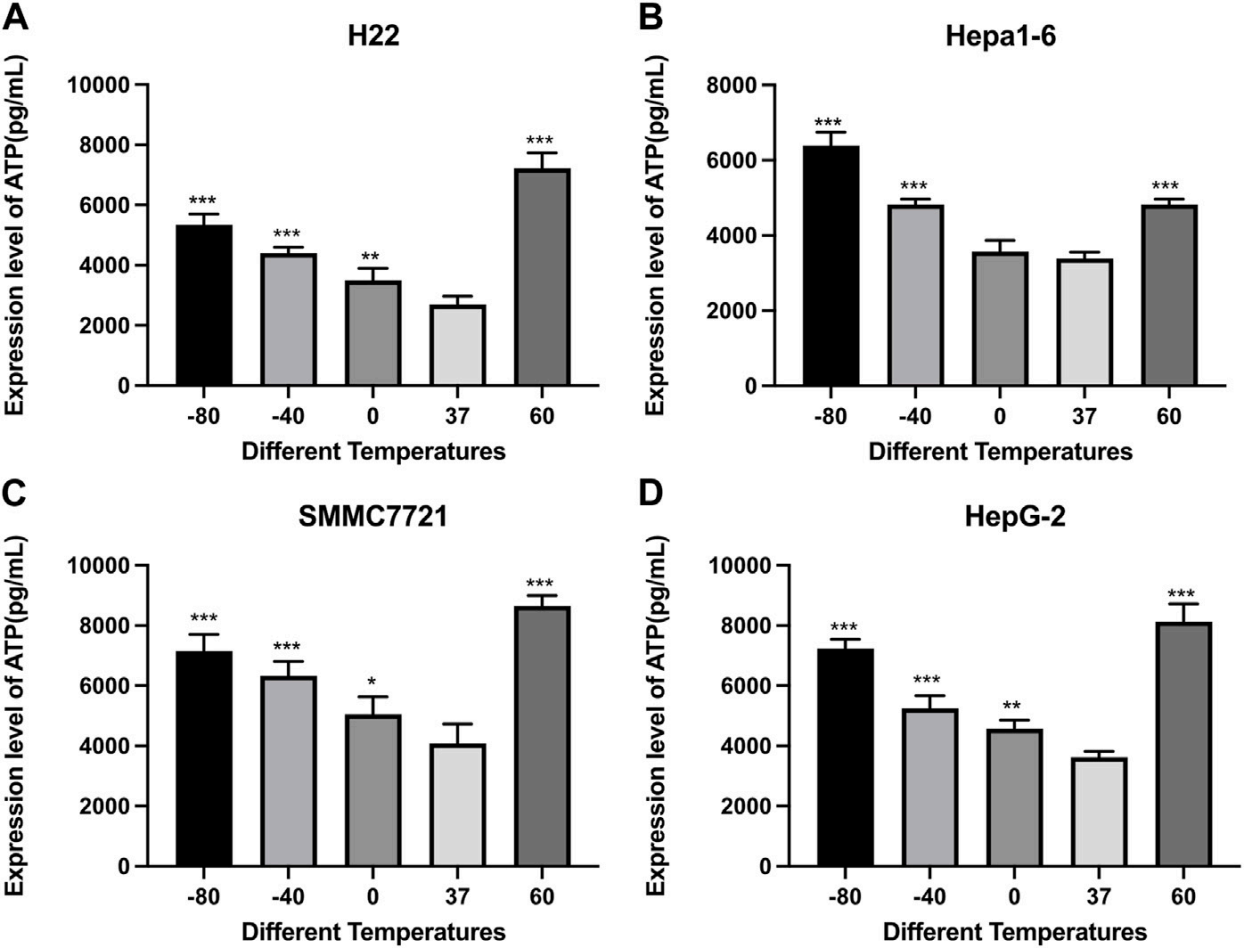
Elisa results of ATP level in cell supernatant of **(A)** H22 cells, **(B)** Hepa1-6cells, **(C)** SMMC7721 cells, and **(D)** HepG2 cells following different thermal ablations (* indicates *p* < 0.05, ** indicates *p* < 0.01 and *** indicates *p* < 0.001).

The ATP and HMGB1 expression levels were detected by commercial ELISA kits. As shown in Figure 4, the concentration of ATP was significantly higher in both cryoablation, and heat ablation groups in comparison to the control group (*p* < 0.01). The increase in ATP concentration in Hepa1-6 cells was greater in −80°C group than in 60°C group (*p* < 0.01). The other three kinds of cells showed higher levels of ATP concentration in 60°C group than in −80°C group (*p* < 0.01).

**FIGURE 5:**
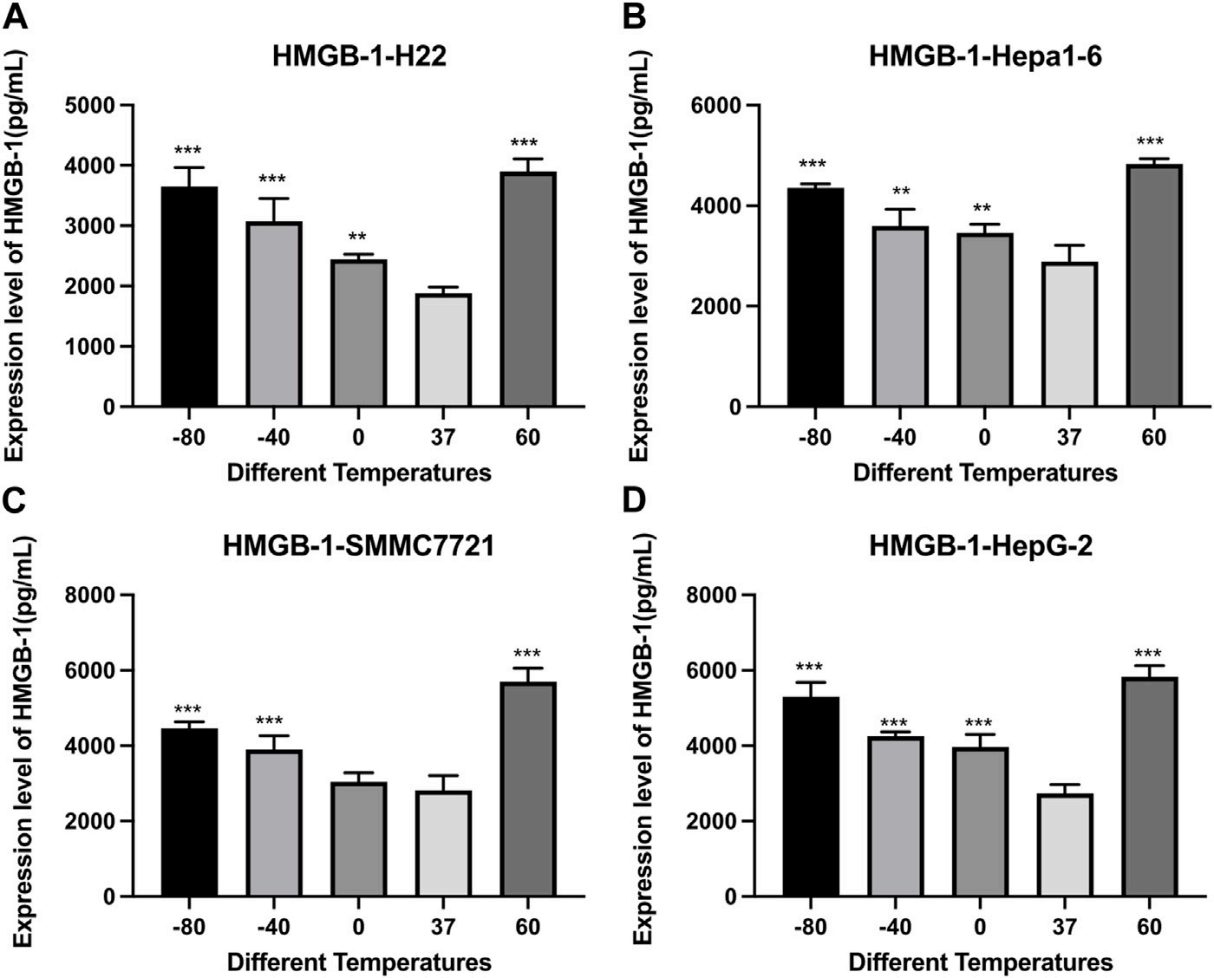
Elisa results of HMGB-1 level in cell supernatant of **(A)** H22 cells, **(B)** Hepa1-6cells, **(C)** SMMC7721 cells, and **(D)** HepG2 cells following different thermal ablations (** indicates *p* < 0.01 and *** indicates *p* < 0.001).

As shown in Figure 5, the concentration of HMGB1 was significantly higher in both cryoablation and heat ablation groups (*p* < 0.01), and the concentration was a little higher in 60°C group than in −80°C group.

### 3.4 Effect of thermal treatment on CXCL10 levels in hepatocarcinoma cells

**FIGURE 6:**
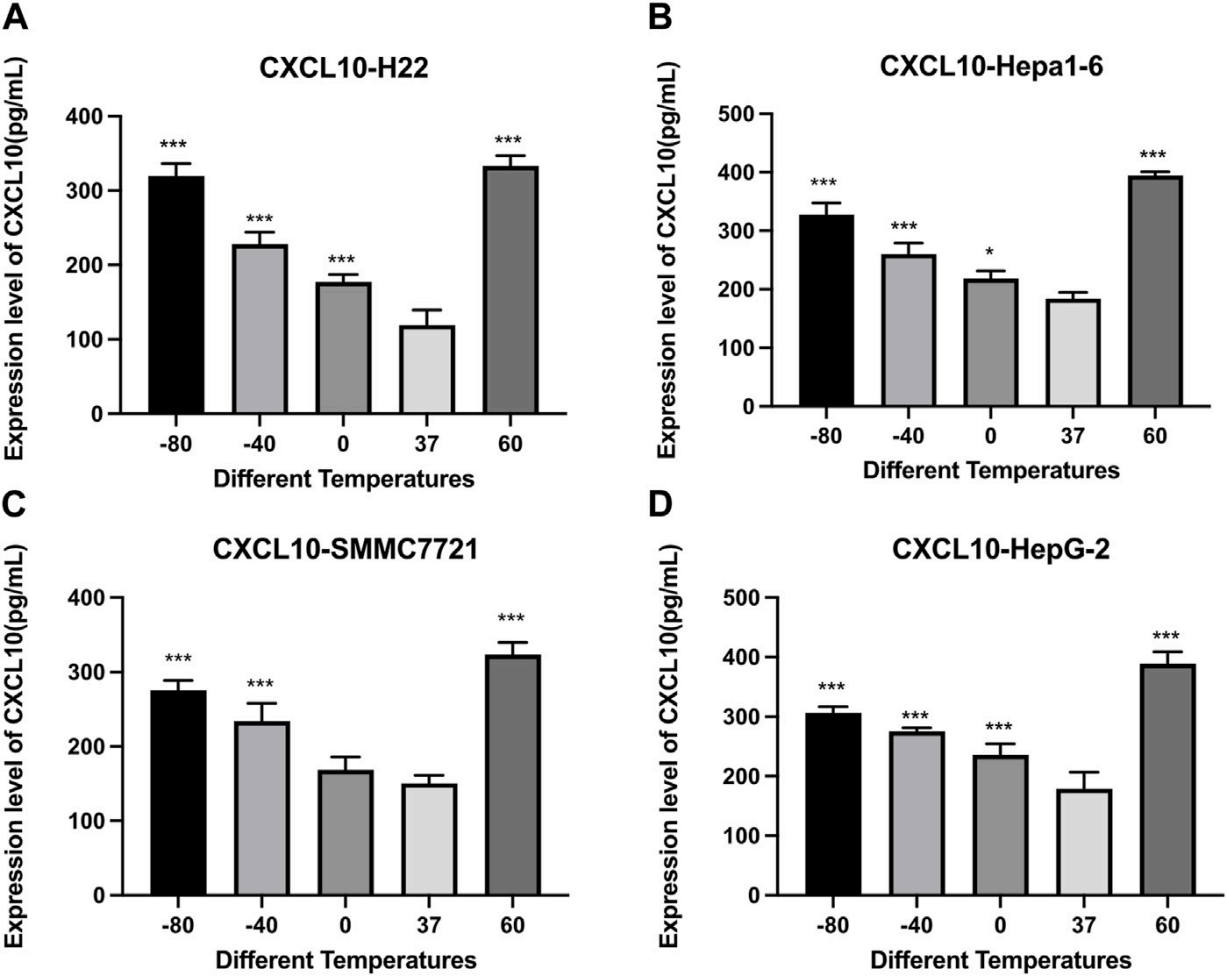
Elisa results of CXCL10 level in cell supernatant of **(A)** H22 cells, **(B)** Hepa1-6cells, **(C)** SMMC7721 cells, and **(D)** HepG2 cells following different thermal ablations (* indicates *p* < 0.05, ** indicates *p* < 0.01 and *** indicates *p* < 0.001).

Figure 6 demonstrates the results of CXCL10 concentrations detected by an Elisa kit. Compared with the control group, the concentrations of CXCL10 increased significantly after cryoablation (*p* < 0.01) and thermal treatment (*p* < 0.01). For H22 and Hepa1-6 cells, the concentrations of CXCL10 were comparable. For SMMC7221 and HepG2 cells, the concentration of CXCL10 was a little higher in 60°C group than in −80°C group.

## 4 Discussion

Distinct from surgery, thermal ablation provokes cellular stress *in situ,* so as to boost the immunogenicity of the tumor by exposing a broad array of neoantigens. In addition, numerous intracellular components that act as co-stimulatory molecules for immune activation are released post-ablation. Increasingly, efforts are being made to integrate the immune system with thermal ablation (Zerbini et al., 2008; Zhang et al., 2008; Skitzki et al., 2009). While ablation may have salutary immunologic properties that enhance tumor immunity, there are also potentially deleterious immunosuppressive and oncogenic sequelae (Sabel, 2009). In patients with prostate cancer, cryoablation may even induce higher or lower levels of regulatory T-cell Treg cells, as well as different functions of Treg (Si et al., 2013). Thus, recognition of these events may form a new basis for improving the efficacy of the combination between conventional therapies and immunotherapies.

ICD is a distinct form of controlled cell death, which is characterized by the secretion of DAMPs in a specific order and can elicit an immune response (Galluzzi et al., 2018). The DAMPs produced by ICD mainly include CRT, HMGB1, ATP, and type 1 IFN, which have a crucial effect in the recruitment and maturation of antigen-presenting cells (APCs) (Fabian et al., 2021; Kroemer et al., 2022). Due to the variability of the cellular stress response activated by each ICD inducer, different ICD inducers generate different DAMPs. ICD parameters could be considered in the development of anticancer therapies (Fucikova et al., 2020). To our knowledge, there were few studies to compare the different DAMPs under different thermal ablation conditions.

Of the existing heat-based ablation modalities (RFA or microwave ablation), 50°C is essentially high enough to cause coagulative necrosis in no time. Cryoablation arrests viability of local cells by removing heat. It has been suggested by a number of studies that temperatures below −20°C can effectively destroy cancer cells. Researchers generally set the lethal temperature at −40°C, although cryoablation can reduce the interstitial temperatures to as low as −160°C. The extent of cellular damage caused by heat-based ablative therapies depends on three factors: the amount of energy applied, the rate of energy delivery, and the target tissue’s intrinsic thermal sensitivity (Mehta et al., 2016). Our study showed that viability of H22 and Hepa16 cells were decreased after thermal treatment or cryoablation, and there was not much difference between −80°C, −40°C, and 60°C group. But for human HCC cells, viability of SMMC7721 cells decreased obviously only in −80°C group, not in −40°C group. And the cell viability (SMMC7721 and HepG2) in −80°C group was much lower than in 60°C group, which indicated that different types of cancer cells have their intrinsic thermal sensitivity.

Within the endoplasmic reticulum, CRT, a soluble protein, carries out several important tasks, which include regulating calcium homeostasis, and assisting in protein folding. When present on the surface of the cell membrane, CRT primarily involves in antigen presentation. CRTs translocated to the membrane of dying cells and distributed in clusters interact with CD91 receptors in phagocytes, which provoke phagocytosis of dying cells (Obeid et al., 2007) and the subsequent tumor antigen cross-presentation and tumor-specific cytotoxic T lymphocyte responses (Kroemer et al., 2013). In this study, H22 cells showed no significant changes in CRT expression under any ablation. Hepa1-6 cells and SMMC7221 cells showed significantly higher CRT expression levels in heat ablation, but lower CRT expression under cryoablation condition. HepG2 cells showed no significant changes in CRT expression levels under heat ablation, but significantly lower CRT expression levels under cryoablation condition.

HMGB1 is a nuclear protein that is abundant in all cell types and functions both inside and outside the cell. As one of the important features of ICD, HMGB1 released in excess to the extracellular space binds to receptors such as Toll-like receptor 4 (TLR4) and receptor for advanced glycation endproducts (RAGE) and promotes the release of more inflammatory cytokines through the activation of NF-κB pathway (Galluzzi et al., 2020). As confirmed by several sources (Cheng et al., 2008; Wu et al., 2016), there is an elevated expression level of HMGB1 in HCC patients, which is inversely correlated with survival. And the role played by HMGB1 and its receptor during HCC carcinogenesis has been described in several articles (Yaser et al., 2012; Chen et al., 2018). In addition, there is *in vitro* evidence for that HMGB1 promotes HCC cell proliferation, migration, and invasion (Xiao et al., 2014). In this study, for H22 cells, Hepa1-6 cells, HepG2 cells, SMMC7221 cells, the concentrations of HMGB1 were significantly higher in both heat ablation and cryoablation condition. And the concentration was a little higher in heat ablation groups. As far as we know, however, HMGB1 exhibits duplicity in tumorigenesis and immunosuppression and may not be considered as a qualified adjuvant for HCC *in situ* vaccine on this account.

It is well known that ATP is the most important energy supplier for all intracellular life activities. Normally, in addition to being present at high levels within the cell, ATP is present at a low level extracellularly. However, under certain stimuli, ATP would be released in excess extracellularly and trigger a series of responses, such as recruitment and transformation of myeloid cells, by binding to and activating purinergic receptor P2Y2 (P2RY2) on the myeloid cell surface (Burnstock, 2007; Elliott et al., 2009). To put it differently, the presence of an adequate amount of extracellular ATP to send “find me” signals and the presence of P2Y family receptors on the surface of myeloid cells are the necessary requirements for ICD (Ghiringhelli et al., 2009). In our study, the concentration of ATP was significantly higher in heat ablation and cryoablation condition, and the concentration was much higher in cryoablation group. Analogous to HMGB1, extracellular release of ATP does not necessarily imply that the ongoing cell death is immunogenic. At least *in vitro*, however, normalizing the level of extracellular ATP to the percentage of dead cells helps to distinguish ICD from other types of cell death.

Chemokines are secreted proteins structurally similar to cytokines and play an important role in regulating the migration of immune cells and the proliferation of tumor cells, including CXCL10, which possess a strong pro-inflammation capacity. Two recent reports have shed light on the connection between CXCL10 and immunotherapy. One of them demonstrated that CXCL10 can be produced not only by immune cells but also by melanoma cells after the application of immunotherapy, which is a prognostic benefit for the tumor (Reschke et al., 2021). The other showed that the combination of MEK inhibitors and chemotherapy promoted the secretion of CXCL10 by cancer cells and the recruitment of tumor-specific cytotoxic T cells (Limagne et al., 2022). In this study, we revealed that ablation therapy was able to induce CXCL10 secretion in all 4 different HCC cell lines. In SMMC7221 and HepG2 cells, the concentration of CXCL10 was a little higher in 60°C group than in −80°C group.

Given the current clinical treatments, the high risk of relapse and acute or chronic liver function impairment after HCC treatment can hardly be addressed. The development of a low-risk and long-term effective treatment for HCC that can fight against the intrinsic immune privilege of liver remains a great challenge, the fulfillment of which depends on further exploration of the immunological features in the tumor microenvironment. In this direction, one of our ultimate goals is to implement an effective vaccine *in situ* that could boost anti-tumor immunity. Solely inducing tumor cell ICDs to release specific antigens is not successful in triggering an effective immune response most of the time. Under immunosuppressive conditions, additional APCs need to be recruited and activated to counteract the relatively insufficient release of DAMPs (Bouzid et al., 2020). As this study showed, different thermal ablation induced different ICD modality in different cancer cells. Precise identification of the different molecular release characteristics of ICDs *in vivo* should accelerate the development of personalized anticancer therapies and identification the optimal combination of treatments for the clinical management of cancer.

Limitations of this study should be mentioned. Firstly, we used water baths and refrigerator storage to simulate heating and cryo-ablation at different temperatures, which did not fully comply with the specifications of clinical ablation operations. Ideally, we should use a needle antenna to deliver energy, and precisely control the temperature. Secondly, our experiments were limited to the *in vitro* level, and no *in vivo* experiments have been performed yet. This is mainly owing to the fact that we could not yet heat or cool the target tissue to a specific temperature *in vivo*. It requires further technological development to break through these limitations.

## Data Availability

The raw data supporting the conclusion of this article will be made available by the author, without undue reservation.
